# *In Vivo* Antimalarial Activity of the Leaf Extract of *Osyris quadripartita* Salzm. ex Decne and Its Major Compound (–) Catechin

**DOI:** 10.1155/2022/3391216

**Published:** 2022-10-07

**Authors:** Teyiba Kemal, Kebede Feyisa, Daniel Bisrat, Kaleab Asres

**Affiliations:** ^1^Department of Pharmacy, College of Health and Medical Science, Haramaya University, Harar, Ethiopia; ^2^Department of Pharmaceutical Chemistry and Pharmacognosy, School of Pharmacy, College of Health Sciences, Addis Ababa University, Addis Ababa, Ethiopia; ^3^Department of Pharmacy, College of Medicine and Health Sciences, Bahir Dar University, Bahir Dar, Ethiopia

## Abstract

**Background:**

The leaves of *Osyris quadripartita* Salzm. ex Decne, endemic to Ethiopia, are traditionally used for the treatment of malaria. Previous phytochemical investigations of *Osyris* species showed the presence of flavonoids, anthracene derivatives, and sesquiterpene lactones as the main constituents. The aim of the present study was to investigate the antimalarial activity of the leaf extract of *O. quadripartita* and its isolated constituent against mice infected with *Plasmodium berghei*.

**Methods:**

Isolation of a compound was carried out on silica gel column chromatography of the extract eluting with gradient mixtures of CHCl_3_/MeOH. Structural elucidation of the isolated compound was achieved by ESI-MS and 1D-and 2D-NMR spectral data. Peter's 4-day suppressive test method was used to determine the antimalarial activity of the test substances. Level of parasitemia, survival time, and body weight change were used to determine the antimalarial activity of the test substances.

**Results:**

(–) Catechin was isolated and characterized from the hydroalcoholic extract of *O. quadripartita*. At a concentration of 400 mg/kg, both the extract and (–) catechin exhibited antimalarial activity with the highest chemosuppression values of 70.61% and 64.26%, respectively.

**Conclusion:**

These findings indicate that *O. quadripartita* is endowed with genuine antimalarial activity attributed in part, to its (–) catechin content. Hence, the present study may validate the traditional use of the plant for the treatment of malaria.

## 1. Introduction

Malaria is an important tropical disease that remains a major health problem in many developing countries. It is a leading cause of hospitalization and one of the world's biggest parasitic killer disease [1, 2]. According to the recent report by the World Health Organization (WHO), an estimated 241 million cases of malaria occurred worldwide in 2020, which led to 627,000 deaths [3]. Africa still bears the largest burden of malaria cases, accounting for 95% of all malaria cases worldwide in 2020 [3]. Despite the high coverage of malaria in Africa, the healthcare facilities are still inadequate, particularly in the rural areas [4]. Children aged under 5 years are at high risk to malaria, accounting for greater than two-thirds of the world malaria deaths [3, 5].

Malaria remains the most serious public health problem in Ethiopia. Over 75% of the population lives in malaria endemic region, exposing more than 50 million people at risk of malaria [6]. A recent report compiled by the Federal Ministry of Health (FMOH) of Ethiopia indicated that out of 1,620,885 suspected cases, 410,409 tested positive for malaria over a period of five years [3, 7].

In Ethiopia, it is estimated that there are more than 7,000 higher plant species of which 12% are endemic [8]. In Ethiopian traditional medicine, more than 200 higher plant species are utilized for the treatment and prevention of malaria [9]. However, scientific studies aimed at validating the genuine antimalarial activities of these plants, which are necessary for new and safe drug development, are very limited.

*Osyris quadripartita* Salzm. ex Decne. belongs to the family Santalaceae, which comprises about 400 species that are partially parasitic on other plants [10–12]. *O. quadripartita* is an evergreen, dioecious tree or shrub which grows up to 1-7 m in height. It has a lot of branches, and the branches are sometimes pendant. In Ethiopia, it is known by several vernacular names including *wato* in Afaan Oromoo and *qeret* in Amharic. It is hemiparasitic and may opportunistically tap into the root systems of adjacent plants, even though it can freely grow and survive [13]. The plant is innate to Africa, southwestern Europe, and Asia [14]. In Ethiopian traditional medicine, it is widely used for the treatment of many diseases including toothache, peptic ulcer disease, cancer, skin lesion, and malaria [13, 15–17]. Previous phytochemical studies have revealed that flavonoids, anthracene derivatives, and sesquiterpene lactones are the main constituents of the leaves and immature fruits of *O. quadripartita* [13, 18, 19]. The present study aimed at evaluating the *in vivo* antiplasmodial activity of the leaf extract of *O. quadripartita* and its major constituent against mice infected with *Plasmodium berghei* [20].

## 2. Materials and Methods

### 2.1. Chemicals and Reagents

Normal phase analytical TLC was performed using silica gel 60 F_254_ precoated plates (0.20 mm) (E-Merck, Darmstadt). Silica gel GF_254_ (UNICHEM^(R)^, India) powder was used for the preparation of self-made 0.5 mm thick preparative TLC (PTLC) plates using glass plates measuring 20 cm × 20 cm. The spot and band were viewed under UV light (254 and 360 nm). Chloroform and methanol were obtained from ReAgent Chemical Services, UK. Solvents were removed using Rota evaporator (BUCHI Rotavapor R-200, Switzerland). ^1^H NMR and ^13^C-NMR spectra were recorded on a Bruker Avance DMX400 FT-NMR spectrometer (Bruker, Billerica, MA, USA). ESI-MS data was generated using a Shimadzu LCMS Advanced spectrometer (Shimadzu, Kyoto, Japan) in the positive-ion mode. Optical rotation was measured using a polarimeter (AUTOPOL®IV, Rudolph Research Analytical, USA).

### 2.2. Plant Materials

Fresh leaves of *O. quadripartita* were collected in February 2019 from Harar Town, East Hararghe zone of Oromia region, about 520 km East of Addis Ababa. The plant was authenticated by Ato Melaku Wondafrash of the National Herbarium, College of Natural and Computational Sciences, Addis Ababa University (AAU), Ethiopia, where a voucher specimen (Collection number TK-001) was deposited.

### 2.3. Extraction of Plant Material

Air-dried powdered leaves of *O. quadripartita* (100 g) were extracted by maceration in 80% methanol for 72 h with occasional shaking. The extract was filtered using a Whatman grade No-1 filter paper (Whatman Ltd, England) and concentrated under reduced pressure a using rotary evaporator to give a yellow brown amorphous substance (24.47 g) [21].

### 2.4. Isolation of Compound

Four fractions were collected when the 80% hydromethanolic extract of *O. quadripartita* (100 g) was subjected to a column chromatography over silica gel eluting with mixtures of CHCl_3_/MeOH gradient (ratio 1 : 0 to 0 : 1). Of these fractions, the second fraction, which was eluted by CHCl_3_/MeOH in a ratio of 3 : 1, yielded white crystals coded OQ-1 (18 mg).

OQ-1 : White crystals; R_*f*_ value of 0.4 (CHCl_3_/CH_3_OH; 3 : 2); [*α*]_*D*_^25^  = −4.0° (*c* = 0.002, EtOH); +ve ESI-MS (Figure S1): *m/z* = 291.20 [M + H]^+^ indicating a relative molecular weight (*M*_*r*_) of 290 (C_15_H_14_O_6_); ^1^H NMR (400 MHz; CD_3_OD; Figure S2): 2.40 (H-4*α*, dd, J = 14.5, 8.6 Hz); 2.75 (H-4*β*, dd, *J* = 14.5, 6.4 Hz); 3.88 (H-3, dd, *J* = 8.6, 6.4 Hz); 4.46 (H-2, *d*, *J* = 8.6 Hz); 5.75 (H-8, *d*, *J* = 1.6 Hz); 5.83 (H-6, *d*, *J* = 1.6 Hz); 6.63 (H-6′, dd, J = 6.4, 1.2 Hz); 6.66 (H-5′, *d*, *J* = 6.4 Hz); 6.73 (H-2′, *d*, *J* = 1.2 Hz). ^13^C-NMR (100 MHz; CD_3_OD; Figure S3): 27.1 (C-4); 67.4 (C-3); 81.5 (C-2); 94.1 (C-8); 94.9 (C-6); 99.4 (C-4a); 113.9 (C-2′); 114.7(C-5′); 118.6 (C-6′); 130.8 (C-1′); 144.8 (C-4′); 144.9 (C-3′)155.5 (C-5); 156.2 (C-8a); 156.5 (C-7). All ^1^H and ^13^C-NMR spectral data were consistent with the one reported for (–) catechin by Seto et al. [22].

### 2.5. Experimental Animals

Healthy Swiss albino mice weighing 20-30 g and aged 6-8 weeks of either sex were obtained from the Animal House of the School of Pharmacy, AAU and kept in plastic cages at room temperature (12 h light/dark cycle). They were fed pellets and water *ad libitum* and were acclimatized for one week under controlled conditions before the commencement of the experiments. All the experiments were carried out in accordance with the internationally accepted laboratory animal use and care guidelines [23].

### 2.6. Acute Oral Toxicity Test

Acute oral toxicity of the test substances (leaf 80% methanol extract and (−) catechin) was carried out according to the OECD guidelines for testing of chemicals on Swiss albino mice [24]. Fifteen healthy Swiss female mice weighing 23-25 g were randomly divided into 3 groups of 5 mice per group. After fasting for 3 h, mice in the first group were given 2 g/kg of the extract, the second group were given 2 g/kg of (−) catechin, and the third group received 0.2 ml distilled water (control group) orally and observed for any signs of toxicity (loss of appetite, hair erection, lacrimation, and mortality) for 14 days to assess safety of the test substances.

### 2.7. Parasite and Preparation of Inoculum

Malaria was induced in the experimental mice using chloroquine-sensitive *Plasmodium berghei* (ANKA) strain. Mice previously infected with *P. berghei* were used as donor. The donor mice were obtained from the Department of Pharmacy, Mekelle University, Mekelle, Ethiopia. The parasites were maintained in the laboratory by sequential blood passage from donor to naive via intraperitoneal injection at interval of 5 days. The parasitemia of the donor mice was determined by preparing blood smears on microscope slides from blood film taken from the tails of infected mice. The smear was fixed with methanol and stained with Giemsa to determine the parasitemia level of the donor under a microscope. When the parasitemia level was 30-40%, parasitized erythrocytes were collected from the donor mouse by cardiac puncture using a sterile syringe tube and were diluted with normal saline to 5 × 10^7^ parasitized erythrocytes per ml. Each mouse was infected by injecting 0.2 ml of this diluted blood via intraperitoneal route [25].

### 2.8. Grouping and Dosing of Animals

Animals were randomly divided into eight groups (six test groups, negative control, and positive control) comprising five mice in each group. The negative control group was treated with distilled water (0.5 ml/kg). Groups II, III, and IV were treated with 100, 200, and 400 mg/kg of the extract, while Groups V, VI, and VII were treated with 100, 200, and 400 mg/kg of (−) catechin, respectively. The positive control group was treated with chloroquine (25 mg/kg) (EPHARM, Ethiopia). Oral doses were determined based on the acute oral toxicity test. All the solutions were freshly prepared on the day of the experiment and administered to mice via oral route using oral gavage for safe ingestion.

### 2.9. 4-Day Suppressive Test

Antimalarial activity of both the extract and isolated compound was evaluated using the methods described by Peter [26]. After inoculation, mice were randomly grouped into eight groups of five mice each and treated as discussed under grouping and dosing of animals. Treatment was started 3 h postinfection of the parasite for each group and then continued for four consecutive days (*D*_0_ to *D*_3_). On the fifth day (*D*_4_), thin smears of blood film taken from the tail of each animal were prepared on three microscopic slides (Sail Brand, China). The smears were applied on microscopic slides and the blood was drawn evenly across a second slide to make a thin blood film and allowed to dry at room temperature. The blood smears were fixed by methanol and stained with 10% Giemsa (Macsen lab, India) for 15 min, and the slides were examined under the microscope (Olympus 6V20WHA2, Japan) with 100× magnifying power using oil immersion.

The parasitemia level was determined by counting the number of parasitized RBCs in random fields of the microscope. Average parasitemia and percent parasitemia suppression were calculated using the following formulas [26].

(1) $$\begin{array}{c} \%\text{Parasitemia}=\left(\frac{\text{Number of parasitized RBC}}{\text{Total number of RBC count}}\right)\;\times 100,\\ \%\text{Suppression}=\left(\frac{\text{Mean parasitemia of negative control}-\text{Mean parasitemia of treated}}{\text{Mean parasitemia of negative control }}\right)\;\times \;100. \end{array}$$

### 2.10. Determination of Mean Survival Time

Mortality was monitored daily, and the number of days from the time of inoculation of the parasite up to death was recorded for each mouse in the treatment and control groups throughout the follow-up period for all test samples. The mean survival time (MST) for each group was then calculated using the following formula:

(2) $$\begin{array}{c} \text{MST}=\left(\frac{\text{Sum of survival time of all mice in a group}\left(\text{days}\right)}{\text{Total number of mice in that group}}\right), \end{array}$$

where MST is mean survival time.

### 2.11. Body Weight Determination

The weight of each mouse in all the groups was measured before infection (*D*_0_) and after treatment (*D*_4_), by using a sensitive electronic balance, and mean body weight changes of the extract and isolated compound treated groups were compared with the control groups [27, 28]. The average body weight change of each treatment group was calculated using the following formula:

(3) $$\begin{array}{c} \text{Average weight change}=\left(\text{Average}D_{4}\text{ of a group}-\text{Average}D_{0}\text{ Weight of that group}\right), \end{array}$$

where *D*_0_ is day zero and *D*_4_ is day 4.

### 2.12. Ethical Approval

All the animal study procedures followed were reviewed and approved by the Institutional Review Board of the School of Pharmacy, College of Health Sciences, Addis Ababa University (approval code: IRB/SOP/092/06/2020). The mice were handled in accordance with the Guide for the Care and Use of Laboratory Animals [23].

### 2.13. Data Analysis

The data are expressed as mean ± SEM (standard error of mean). One-way ANOVA was used to analyze the differences between the means among groups followed by Tukey post hoc test to compare subgroup. The differences were considered statistically significant if *p* < 0.01. The data were analyzed using Windows SPSS Version 25.

## 3. Results

### 3.1. Structural Elucidation of the Isolated Compound

A flavanol was isolated from the hydromethanolic leaf extract of *O. quadripartita* by column chromatography over silica gel eluting with CHCl_3_/CH_3_OH gradients. The compound was obtained as a white crystal with a specific optical rotation of [*α*]_*D*_^25^  = −4.0° (*c* = 0.002, EtOH) and a retention factor (R_*f*_) value of 0.4 in CHCl_3_/CH_3_OH (3 : 2) solvent system. The positive-mode electrospray ionization mass spectrum (ESI-MS; Figure S1) of the compound gave a pseudomolecular ion peak at *m/z* 291.20 [M + H]^+^, corresponding to a relative molecular weight (*M*_*r*_) of 290 amu. Its molecular formula was deduced as C_15_H_14_O_6_ on the basis of ESI-MS and ^1^H and ^13^C-NMR spectral data.

**Figure 1:**
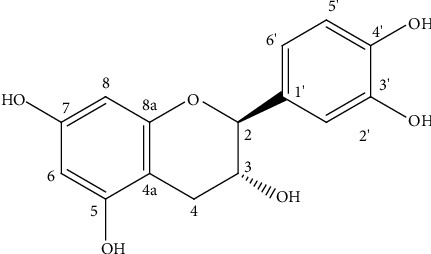
Chemical structure of (−) catechin.

^1^H NMR spectrum (Figure S2) exhibited proton signals of a typical flavan-3-ol nucleus, which were indicated by five aromatic methines at *δ* 5.75 (1H, *d*, *J* = 1.6 Hz, H-8), 5.83 (1H, *d*, *J* = 1.6 Hz, H-6), *δ* 6.63 (1H, dd, *J* = 6.4, 1.2 Hz, H-6′), *δ* 6.66 (1H, *d*, *J* = 6.4 Hz, H-5′), and *δ* 6.73 (1H, *d*, *J* = 1.2 Hz, H-2′), two nonequivalent protons in the dihydropyran ring at *δ* 2.40 (1H, dd, *J* = 14.5, 8.6 Hz, H-4*α*) and *δ* 2.75 (1H, dd, *J* = 14.5, 6.4 Hz, H-4*β*), two oxymethines at *δ* 4.46 (1H, *d*, *J* = 8.6 Hz, H-2), and a doublet of doublet at *δ* 3.88 (1H, dd, *J* = 8.6, 6.4 Hz, H-3). The ^13^C-NMR spectrum (Figure S3) revealed 15 carbon signals, which were further grouped to a methylene group (*δ* 27.1), five aromatic methines (*δ* 94.1, *δ* 94.9, *δ* 113.9, *δ* 114.7, and *δ* 118.6), and seven quaternary carbons (*δ* 99.4, *δ* 130.8, *δ* 144.9, *δ* 144.8, *δ* 155.5, *δ* 156.2, and *δ* 156.5) by DEPT-135 (Figure S4) and HSQC spectral data. Therefore, the compound was unequivocally identified as (−) catechin (Figure 1) by comparing its ^1^H and ^13^C-NMR spectra and optical rotation data with the same compound reported by Nonaka et al. [29].

### 3.2. Acute Oral Toxicity Test

Both the hydroalcoholic leaf extract and (−) catechin were found to be safe at a dose of 2000 mg/kg as no mortality or any signs of gross physical and behavioral changes were observed within the first 24 h and the following 14 days. Therefore, oral LD_50_ of the leaf extract and (−) catechin is well above 2000 mg/kg.

### 3.3. In Vivo Antimalarial Activity

In this study, the in vivo antimalarial activity of both the hydroalcoholic leaf extract and (−) catechin was determined using the 4-day suppressive test to evaluate the chemosuppressive, survival time, and body weight loss effects of the test substances [26].

As indicated in Table 1, the hydroalcoholic leaf extract showed a significant chemosuppressive effect in mice infected with *P. berghei* in a dose-dependent manner, ranging from 54.69 to 70.61% suppression as compared to the negative control. This is in contrary to a report by Girma et al. [14], who stated that all tested doses of the methanol leaf extract of *O. quadripartita*, except the 200 mg/kg, have exhibited a significant chemosuppressive activity against *P*. *berghei*. This difference in potency might be due to the natural habitat of the plant and collection time. (−) Catechin also produced a significant chemosuppression in a dose-related manner. There was a significant decline in parasitemia (*p* < 0.01) of 100, 200, and 400 mg/kg (−)catechin-treated groups when compared to the negative control.

An antimalarial agent is considered active when it causes parasitemia suppression 30% or more [30]. The increased percent suppression of parasitemia with increased dose was also observed in other studies conducted on other plants [1, 31]. Average parasite load at the doses employed was lower than that observed in the untreated mice. The increased percent suppression of parasitemia with increased dose was also observed in other studies conducted on other plants [1, 31]. The standard drug chloroquine that was used as control drug resulted in 99.6% eradication of the parasite at a concentration of 25 mg/kg (Table 1).

Mean survival time of *P. berghei* infected mice is another parameter to evaluate the antimalarial activity of a test substance [1, 32]. As shown in Table 1, comparison among the test substance dose levels showed that at 400 mg/kg both the hydroalcoholic extract and (–)-catechin significantly prolonged (*p* < 0.01) survival time when compared to vehicle treated group. This might have happened due to their suppressive effect on growth of the parasite. However, the mean survival times of test substances treated mice at all doses were shorter than those treated with chloroquine. This might be due to recrudescence of the disease which results in early death [33] due to shorter half-life of the active constituents in the plasma [34].

### 3.4. Effect on Weights of Mice

Body weight loss is one feature of rodent malaria infection [4]. It is caused due to appetite depressant action of the parasite on mice and the disturbed metabolic function and hypoglycemic effect of the parasite [10, 35]. Results of the present study revealed that the hydroalcoholic extract significantly (*p* < 0.01) increased the body weight of the infected mice at doses of 100, 200, and 400 mg/kg as compared to the negative control. Similarly, at all doses employed, (−) catechin significantly (*p* < 0.01) increased body weight of the experimental animals compared to the negative control (Table 2). Body weight increase among the experimental animals is an indication that the test substances might have effects other than direct parasiticidal effect. Some antimalarial plants have shown other pharmacologic benefits to the host: like acting as analgesics, antipyretics, immune stimulators, or may contain appetite-enhancing agent(s) [36]. Previous studies have shown that the extracts of *O. quadripartita* possess antioxidant [37], antibacterial, and antifungal [38] activities.

## 4. Discussion

Catechins are polyphenol flavanols found abundantly in many plants and previously isolated from a number of medicinal plants such as *Osyris alba* [39], *Camellia sinensis* leaves [40], *Trichilia emetica* whole seeds [41], *Acacia catechu* [42], aerial parts of *Astragalus glycyphyllos* [43], and others. Catechin has been shown to have various pharmacological activities such as antioxidant [44], anti-inflammatory [45], and antiparasitic [39]. Paveto et al. [40] demonstrated that catechins possess strong lytic activity on bloodstream trypomastigotes. Similarly, a crude extract of green tea and its two main constituents, epigallocatechin-3-gallate and epicatechin gallate, were shown to inhibit *Plasmodium falciparum* growth *in vitro* [46]. It was found that the antioxidant activity of green tea catechins correlates to antimalarial property, especially the interference with fatty acid biosynthesis may represent a primary mechanism [46].

Catechins exist in four different stereoisomers. Previously, Abdulah et al. [47] reported that (+)-catechin isolated from the leaves of *Garcinia celebica* possesses *in vitro* growth inhibitory effect against the trophozoite and schizont stages *P*. *falciparum*. The current study has demonstrated that (−) catechin like other dietary flavonoids such as myricetin, quercetin, apigenin, and luteolin which are known to inhibit the intraerythrocytic growth of the 3D7 and 7G8 strains of *P. falciparum* [48] possesses antimalarial activity. The mechanism of antiplasmodial action of (−) catechin has not been elucidated. However, plausible mechanisms by which the compound exerts its action could be proposed based on its structural features. Perusal of literature reveals that the antiplasmodial activity of flavonoids is due to their antioxidant property [49]. Moreover, it is believed that flavonoids act by inhibiting fatty acid biosynthesis (FAS II) of the parasite [50] and bind parasite's serine threonine kinase with high affinity and affect its development [51]. As catechin has phenolic OH groups, it may exert its antiplasmodial action by one or a combination of the above mechanisms.

## 5. Conclusions

The present findings clearly showed that the hydroalcoholic leaf extract of *O*. *quadripartita* and (−) catechin exerts a significant dose-dependent chemosuppression effect in mice infected with *P. berghei*. Therefore, it stands to reason that the antimalarial activity of *O*. *quadripartita* leaves is attributed in part to the presence of (−) catechin. Although (−) catechin caused significant suppression in parasite count, its effect was somewhat inferior to that of the hydroalcoholic extract suggesting that the plant contains other active compounds which exert synergistic therapeutic effects. Even though neither the extract nor the isolated compound was as active as chloroquine, the study upholds the traditional claim of the plant for the treatment of malaria. However, further studies are needed to elucidate the mechanism of action of the test substances.

## Figures and Tables

**Table 1:** Percentage suppression and mean survival time of *Plasmodium berghei*-infected mice after administration of 80% methanol leaf extract of *Osyris quadripartita* and its constituent (−) catechin.

Treatment	Dose (mg/kg, p.o)	% Parasitemia ± SEM	% Suppression	Survival time (in days) ± SEM
Vehicle	0.5 ml	49.00 ± 1.92	0.00^a^	5.60 ± 0.40^a^

Leaf extract	100	22.20 ± 1.15	54.69^b^	10.80 ± 0.37^b^
	200	18.60 ± 0.92	62.04^b,c^	14.00 ± 0.70^c^
	400	14.40 ± 1.12	70.61^c^	14.60 ± 0.51^c^

(–) Catechin	100	27.60 ± 0.24	42.55^d^	10.60 ± 0.51^d^
	200	21.40 ± 0.51	54.47^e^	11.80 ± 0.66^d^
	400	16.80 ± 0.37	64.26^f^	12.60 ± 0.51^d^

Chloroquine	25	0.20 ± 0.19	99.60^g^	30.20 ± 1.77^e^

*Note.* Values are presented as mean ± SEM; *n* = 5; means followed by a different letter indicate significant differences between different doses of the same treatments, vehicle, and positive control in the same column (*p* < 0.01).

**Table 2:** Body weight of *Plasmodium berghei*-infected mice after administration of the 80% methanol leaf extract of *Osyris quadripartita* and (−) catechin.

Treatment	Dose (mg/kg, p.o)	Wt.(g) *D*_0_ ± SEM	Wt.(g) *D*_4_ ± SEM	Weight change
Vehicle	0.5 ml	24.80 ± 1.50	23.00 ± 1.73	−7.26^a^

Leaf extract	100	27.20 ± 0.80	28.20 ± 0.73	3.70^b^
	200	24.80 ± 0.86	26.20 ± 0.86	5.60^b^
	400	25.00 ± 0.55	26.00 ± 0.55	4.00^b^

(–) Catechin	100	22.80 ± 0.97	23.80 ± 0.66	4.39^c^
	200	23.00 ± 1.00	23.60 ± 0.60	2.61^c^
	400	22.00 ± 1.58	23.20 ± 1.24	5.45^c^

Chloroquine	25	29.20 ± 1.39	31.80 ± 1.36	8.90^d^

*Note.* Values are presented as mean ± SEM; *n* = 5; means followed by a different letter indicate significant differences between different doses of the same treatments, vehicle, and positive control in the same column (*p* < 0.01).

## Data Availability

The data used to support the findings of this study are available from the corresponding author upon request.

## Supplementary Materials

Supplementary Materials Figure S1 : positive-mode electrospray ionization mass spectrum (ESI-MS (+-mode)) of OQ-1. Figure S2 : ^1^H NMR spectrum of OQ-1. (A) : full ^1^H NMR spectrum (0-7.00 ppm); (B) : expanded-^1^H NMR spectrum (6.55-6.75 ppm); (C) : expanded-^1^H NMR spectrum (5.70-5.85 ppm). Figure S3 : ^13^C-NMR spectrum of OQ-1. Figure S4 : DEPT-135 spectrum of OQ-1.
