# Supplementary figures and images for: In Vivo Antimalarial Activity of the Leaf Extract of Osyris quadripartita Salzm. ex Decne and Its Major Compound (–) Catechin
Source: J Trop Med. 2022 Oct 7;2022:3391216. doi: 10.1155/2022/3391216 (PMC9568338; doi:10.1155/2022/3391216)

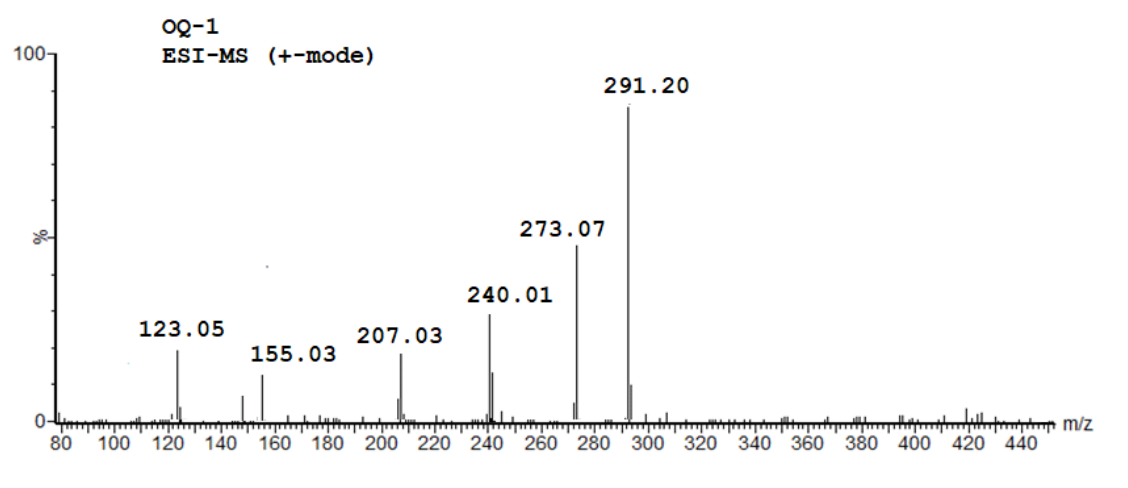


FIGURE S1: Positive-mode electrospray ionization mass spectrum (ESI-MS (+-mode)) of OQ-1

Supplement: Supplementary Materials — Figure S1 : positive-mode electrospray ionization mass spectrum (ESI-MS (+-mode)) of OQ-1. Figure S2 : 1H NMR spectrum of OQ-1. (A) : full 1H NMR spectrum (0-7.00 ppm); (B) : expanded-1H NMR spectrum (6.55-6.75 ppm); (C) : expanded-1H NMR spectrum (5.70-5.85 ppm). Figure S3 : 13C-NMR spectrum of OQ-1. Figure S4 : DEPT-135 spectrum of OQ-1. [file 3391216.f1.zip › FIGURE S1.docx]
